# Exploring artificial intelligence in orthopaedics: A collaborative survey from the ISAKOS Young Professional Task Force

**DOI:** 10.1002/jeo2.70181

**Published:** 2025-02-24

**Authors:** Filippo Familiari, Adnan Saithna, Juan Pablo Martinez‐Cano, Jorge Chahla, Juan Miguel Del Castillo, Nicholas N. DePhillipo, Gilbert Moatshe, Edoardo Monaco, Jaime Palos Lucio, Pieter D'Hooghe, Robert F. LaPrade, the ISAKOS Young Professionals Task Force

**Affiliations:** ^1^ Department of Orthopaedic and Trauma Surgery Magna Graecia University Catanzaro Italy; ^2^ Research Center on Musculoskeletal Health (MusculoSkeletalHealth@UMG) Magna Graecia University Catanzaro Italy; ^3^ Department of Orthopedic Surgery University of Arizona Tucson Arizona USA; ^4^ AZBSC Orthopedics Scottsdale Arizona USA; ^5^ Departamento de Ortopedia Fundación Valle del Lili Cali Colombia; ^6^ Universidad Icesi Cali Colombia; ^7^ Department of Orthopaedic Surgery Rush University Medical Center Chicago Illinois USA; ^8^ Midwest Orthopaedics at Rush Chicago Illinois USA; ^9^ Clínica de Traumatología y Ortopedia Universidad de la República Montevideo Uruguay; ^10^ Department of Orthopedics University of Pennsylvania Philadelphia Pennsylvania USA; ^11^ Oslo Sports Trauma Research Center Norwegian School of Sports Science Oslo Norway; ^12^ Orthopaedic Clinic Oslo University Hospital Ullevål Oslo Norway; ^13^ Orthopaedic Unit University of Rome La Sapienza, Sant'Andrea Hospital Rome Italy; ^14^ Department of Orthopedic Surgery Hospital Central Dr Ignacio Morones Prieto San Luis Potosí Mexico; ^15^ Hospital Lomas de San Luis International San Luis Potosí Mexico; ^16^ Aspetar Orthopaedic and Sports Medicine Hospital Doha Qatar; ^17^ Twin Cities Orthopedics Edina Minnesota USA

**Keywords:** arthroscopy, artificial intelligence, diagnosis, orthopaedics, sports medicine

## Abstract

**Purpose:**

Through an analysis of findings from a survey about the use of artificial intelligence (AI) in orthopaedics, the aim of this study was to establish a scholarly foundation for the discourse on AI in orthopaedics and to elucidate key patterns, challenges and potential future trajectories for AI applications within the field.

**Methods:**

The International Society of Arthroscopy, Knee Surgery and Orthopaedic Sports Medicine (ISAKOS) Young Professionals Task Force developed a survey to collect feedback on issues related to the use of AI in the orthopaedic field. The survey included 26 questions. Data obtained from the completed questionnaires were transferred to a spreadsheet and then analyzed.

**Results:**

Two hundred and eleven orthopaedic surgeons completed the survey. The survey encompassed responses from a diverse cohort of orthopaedic professionals, predominantly comprising males (92.9%). There was wide representation across all geographic regions. A notable proportion (52.1%) reported uncertainty or lack of differentiation among AI, machine learning and deep learning (47.9%). Respondents identified imaging‐based diagnosis (60.2%) as the primary field of orthopaedics poised to benefit from AI. A considerable proportion (25.1%) reported using AI in their practice, with primary reasons including referencing scientific literature/publications (40.3%). The vast majority expressed interest in leveraging AI technologies (95.3%), demonstrating an inclination towards incorporating AI into orthopaedic practice. Respondents indicated specific areas of interest for further study, including prediction of patient outcomes after surgery (30.8%) and image‐based diagnosis of osteoarthritis (28%).

**Conclusions:**

This survey demonstrates that there is currently limited use of AI in orthopaedic practice, mainly due to a lack of knowledge about the subject, a lack of proven evidence of its real utility and high costs. These findings are in accordance with other surveys in the literature. However, there is also a high level of interest in its use in the future, in increased study and further research on the subject, so that it can be of real benefit and make AI an integral part of the orthopaedic surgeon's daily work.

**Level of Evidence:**

Level IV, survey study.

**Abbreviations**
AI: artificial intelligence
ChatGPT: Chat Generative Pre‐trained Transformer
DL: deep learning
ISAKOS: International Society of Arthroscopy, Knee Surgery and Orthopaedic Sports Medicine
ISHKS: Indian Society of Hip and Knee Surgeons
ML: machine learning
OA: osteoarthritis

## INTRODUCTION

In recent years, the convergence of artificial intelligence (AI) and orthopaedic surgery has emerged as a focal point for transformative research and technological innovation [5, 8]. The intricacies and demand for precision within orthopaedic surgery render it a field poised to greatly benefit from AI applications [4, 14]. AI has the potential to enhance these aspects by providing advanced imaging analysis, predictive modelling, and decision support systems. AI‐driven predictive models can assist in preoperative planning by simulating surgical outcomes based on patient‐specific data, thereby optimizing surgical strategies and clinical capabilities, ultimately leading to more precise diagnoses, personalized treatment plans and enhanced surgical outcomes. Acknowledging the imperative to comprehensively understand the current landscape of AI integration, the International Society of Arthroscopy, Knee Surgery and Orthopaedic Sports Medicine (ISAKOS) initiated a survey through its Young Professionals Task Force.

The integration of AI technologies in medical settings has shown promise in augmenting diagnostic accuracy, optimizing therapeutic interventions and refining surgical approaches [9]. In orthopaedic surgery, where precision and efficiency are paramount, the potential impact of AI on clinical outcomes is of particular interest. While existing literature has explored AI applications in broader medical contexts [1], a comprehensive understanding of its current status, challenges and opportunities within the specific domain of orthopaedic surgery has yet to be reported.

The ISAKOS Young Professionals Task Force's survey was meticulously designed to address this gap in knowledge. Targeting a diverse cohort of young professionals in orthopaedic surgery, the survey aims to capture a broad spectrum of perspectives and experiences related to AI adoption. Leveraging the international scope of ISAKOS, this initiative ensures a global representation, facilitating a comprehensive examination of regional variations in AI integration practices and challenges.

Through an analysis of findings from a survey about the use of AI in orthopaedics, the aim of this study was to establish a scholarly foundation for the discourse on AI in orthopaedics and to elucidate key patterns, challenges and potential future trajectories for AI applications within the field.

## MATERIALS AND METHODS

The ISAKOS Young Professionals Task Force developed a survey with the goal to collect feedback on issues in the use of AI in the orthopaedic field. The 26‐question multiple choice survey was a self‐administered questionnaire in English that was built using Google Forms, a free open‐source software. The survey was sent via e‐mail on 17 February 2023. Three email reminders for follow‐up surveys were sent, and the first reminder was two weeks after the initial one. Within the e‐mail, the invitees were provided with a brief explanation of the purpose of the survey and were asked to click on a link that would lead them to the appropriate version of the survey. The survey required approximately 5–10 min to complete. The survey needed to be brief to maximize the response rate. The survey was closed on 31 March 2023. Anonymous data obtained from the completed questionnaires were transferred in a spreadsheet and then analyzed using Microsoft Excel (Microsoft Office 365 for Windows) [10]. The questions are listed in Table 1 with the specific multiple‐choice answers listed in Tables 2, 3, 4, 5 in the Results section. Questions 1–7 questions targeted demographic information, Questions 6–13 were about the use of AI, Questions 14–21 targeted definitions and applications of AI, machine learning (ML) and deep learning (DL) in medicine and orthopaedics, and Questions 22–26 were about future perspectives in AI.

**Table 1:** The 26‐question survey.

1.	Gender
2.	Are you currently an ISAKOS member?
3.	In which geographic region do you reside?
4.	Choose your profession
5.	Provide your years of experience
6.	Choose your primary professional activity
7.	Choose your type of practice*
8.	Do you use artificial intelligence AI in your practice?
9.	What are your primary reasons for choosing artificial intelligence?*
10.	Do you routinely use AI/ML/DL for imaging‐based diagnosis?
11.	Do you routinely use AI/ML/DL as intraoperative aid?
12.	Do you routinely use AI/ML/DL for robotic surgery?
13.	What do you think about the use of Big Data in your practice?
14.	Do you know the difference among AI, ML and DL?
15.	Do you agree with the following definition of AI: It involves machines that can perform tasks innately characteristic of human intelligence (planning, understanding language, recognizing patterns, learning and problem solving) and it can learn from mistakes and improve, which resembles experiential learning
16.	Do you agree with the following definition of ML: ML algorithms are able to learn from examples by adjusting their internal parameters (weights) and strengthening relevant associations to improve the accuracy of a given model
17.	Do you agree with the following definition of DL: DL is a more sophisticated form of ML. DL is capable of unsupervised learning from unstructured and unlabelled inputs and filtering out data input from variables of low relevance to the prediction of interest. DL is modelled after the brain's neuronal connections via algorithms termed artificial neural networks (ANNs)
18.	Do you know where and when AI/ML/DL are used in Medicine?
19.	Do you know where and when AI/ML/DL are used in Orthopaedic Surgery?
20.	Do you know how many systems using Big Data are currently available?
21.	In which field of Orthopaedic Surgery do you think that AI would be more beneficial?
22.	ML/DL should be studied more for automated detection, classification, and prediction of what kind of osteoarticular pathologies in your opinion?
23.	Which is the best application for AI/ML/DL?
24.	Are you interested in using artificial intelligence in the future?
25.	Which artificial intelligence technology would you like to use most in the future?
26.	What are the main reasons NOT to use artificial intelligence at this point in time?*

*Note*: In questions marked with (*), more than one option can be answered.

Abbreviations: AI, artificial intelligence; DL, deep learning; ISAKOS, International Society of Arthroscopy, Knee Surgery, and Orthopaedic Sports Medicine; ML, machine learning.

**Table 2:** Study participants.

Variable		Absolute frequency	Relative frequency
Gender	Female	15	7.1%
	Male	196	92.9%
ISAKOS member	Yes	166	78.7%
	No	39	18.5%
	Not sure	6	2.8%
Geographic region	Africa‐Middle East	17	8%
	Asia‐Pacific	54	25.6%
	Europe	80	37.9%
	Latin America	32	15.2%
	North America	28	13.3%
Profession	Medical doctor	206	97.6%
	Physical therapist	1	0.5%
	Researcher	3	1.4%
	Student	1	0.5%
Years of experience	Less than 5 years	33	15.6%
	5–10 years	32	15.2%
	11–20 years	35	16.6%
	More than 20 years	111	52.6%
Primary professional activity	Adult reconstructive surgery	15	7.1%
	Orthopaedic surgery	133	63%
	Sports medicine	56	26.5%
	Trauma	7	3.3%
Type of practice a	Private	131	45%
	Public	68	23.4%
	Academic	91	31.3%
	Emeritus	1	0.3%

Abbreviation: ISAKOS, International Society of Arthroscopy, Knee Surgery, and Orthopaedic Sports Medicine.

^a^ More than one option could be answered.

**Table 3:** Understanding and awareness of AI.

Variable		Absolute frequency	Relative frequency
Difference among AI, ML and DL	Yes	101	47.9%
	No	110	52.1%
Definition of AI	Yes	191	90.5%
	Not sure	4	1.9%
	No	13	6.2%
Definition of ML	Yes	186	88.2%
	Not sure	6	2.8%
	No	14	6.6%
Definition of DL	Yes	172	81.5%
	Not sure	13	6.2%
	No	19	9%
Know where and when AI, ML and DL are used in medicine	Yes	86	40.8%
	Partially/not clear	9	4.3%
	No	113	53.6%
Know where and when AI, ML and DL are used in orthopaedics	Yes	79	37.4%
	Partially/not clear	14	6.6%
	No	115	54.5%
How many systems of Big Data are currently available	0	63	29.9%
	1	25	11.9%
	2	19	9%
	>2	91	43.1%
	No answer	13	6.2%
Field of orthopaedics to benefit from AI	Imaging‐based diagnosis	127	60.2%
	Advancement of value‐based care	69	32.7%
	Others	11	5.2%
	No answer	4	1.9%

Abbreviations: AI, artificial intelligence; DL, deep learning; ML, machine learning.

**Table 4:** Use of AI.

Variable		Absolute frequency	Relative frequency
Use of AI in practice	Yes	53	25.1%
	No	158	74.9%
Primary reasons for choosing AI a	Meeting presentations	62	19.2%
	Scientific literature/publications	130	40.3%
	Improving surgical outcomes	4	1.2%
	Personal communications	50	15.5%
	Previous experience with AI	56	17.3%
	Others	21	6.5%
Routinely use AI/ML/DL for image‐based diagnosis	Always	4	2%
	Often	21	10%
	Rarely	48	22.8%
	Never	135	64%
Routinely use AI/ML/DL for intraoperative aid	Always	5	2.4%
	Often	20	9.5%
	Rarely	34	16.1%
	Never	149	70.6%
Routinely use AI/ML/DL for robotic surgery	Always	7	3.3%
	Often	27	12.8%
	Rarely	25	11.9%
	Never	147	69.7%
Thinking about usage of Big Data	Improve diagnosis	64	30.3%
	Improve treatment	100	47.4%
	Neither benefit nor disadvantage	34	16.1%
	Worsen diagnosis	2	1%
	Worsen treatment	2	1%

Abbreviations: AI, artificial intelligence; DL, deep learning; ML, machine learning.

^a^ More than one option could be answered.

**Table 5:** AI and its future.

Variable		Absolute frequency	Relative frequency
Interested in using AI in the future	Yes	201	95.3%
	No	8	3.8%
Should ML/DL be studied more	Identification of arthroplasty implants	28	13.3%
	Image‐based diagnosis of OA degenerative disease	59	28%
	Image‐based diagnosis of OA traumatic disease	27	12.8%
	Patient monitoring	15	7.1%
	Prediction of patient outcomes after surgery	65	30.8%
	Prediction of post‐operative complications	13	6.2%
Which AI technology would like the most in the future	AI	128	60.7%
	DL	44	20.9%
	ML	32	15.2%
Main reasons NOT to use AI at this point in time^a^	Country‐specific regulatory issues	33	12.5%
	High cost for low value	79	29.8%
	No evidence of efficacy established	64	24.2%
	No information available	80	30.2%
	Others	9	3.4%

Abbreviations: AI, artificial intelligence; DL, deep learning; ML, machine learning; OA, osteoarthritis.

^a^ More than one option could be answered.

## RESULTS

### Study participants

**Figure 1:**
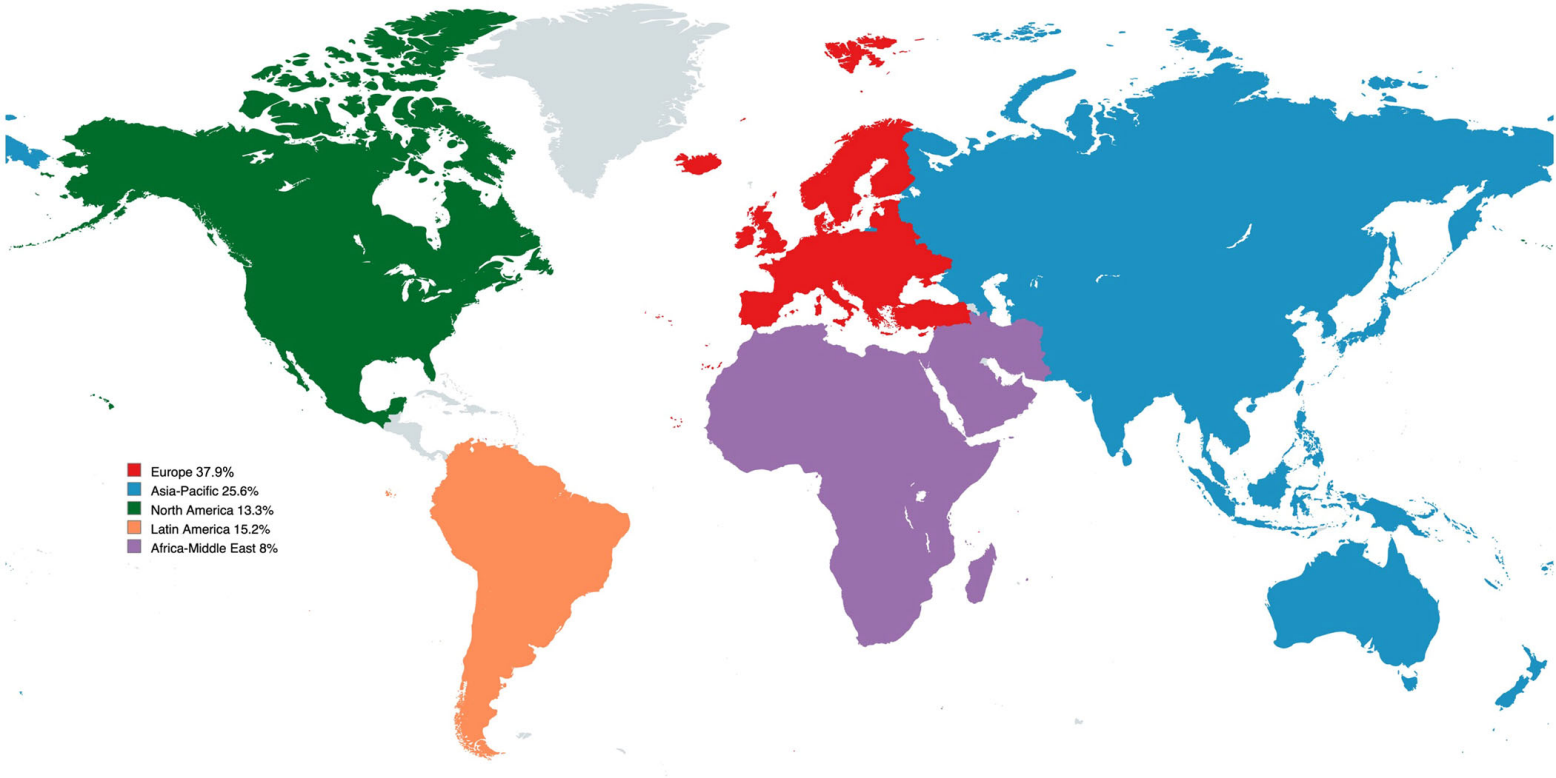
Distribution of survey participants for artificial intelligence worldwide.

Two hundred and eleven orthopaedic surgeons completed the survey. The survey encompassed responses from a diverse cohort of orthopaedic professionals, predominantly comprising males (92.9%). There was varying representation across geographic regions: Europe (37.9%), Asia‐Pacific (25.6%), Latin America (15.2%), North America (13.3%) and Africa‐Middle East (8%) (Figure 1). Notably, over half (52.6%) of the respondents reported more than 20 years of experience. Orthopaedic surgery (63%) emerged as the predominant professional activity, followed by sports medicine (26.5%) and adult reconstructive surgery (7.1%). Practice settings varied, with a vast proportion in private practice (45%), followed by academic (31.3%) and public (23.4%) sectors. Results are shown in Table 2.

### Understanding and awareness of AI

A vast majority exhibited awareness of the distinctions among AI, ML and DL (47.9%); however, a notable proportion (52.1%) reported uncertainty or lack of differentiation. Regarding definitions, the majority demonstrated understanding of AI (90.5%), ML (88.2%) and DL (81.5%). In terms of awareness of AI, ML and DL applications in medicine, a sizable percentage reported partial or a lack of understanding (53.6%). Similarly, in orthopaedics, awareness was limited, with the majority reporting partial understanding or lack of clarity (54.5%). Regarding big data systems, participants exhibited varied knowledge, with a vast proportion reporting the availability of more than two systems (43.1%). Finally, respondents identified imaging‐based diagnosis (60.2%) as the primary field of orthopaedics poised to benefit from AI, followed by the advancement of value‐based care (32.7%). Results are shown in Table 3.

### Use of AI

**Figure 2:**
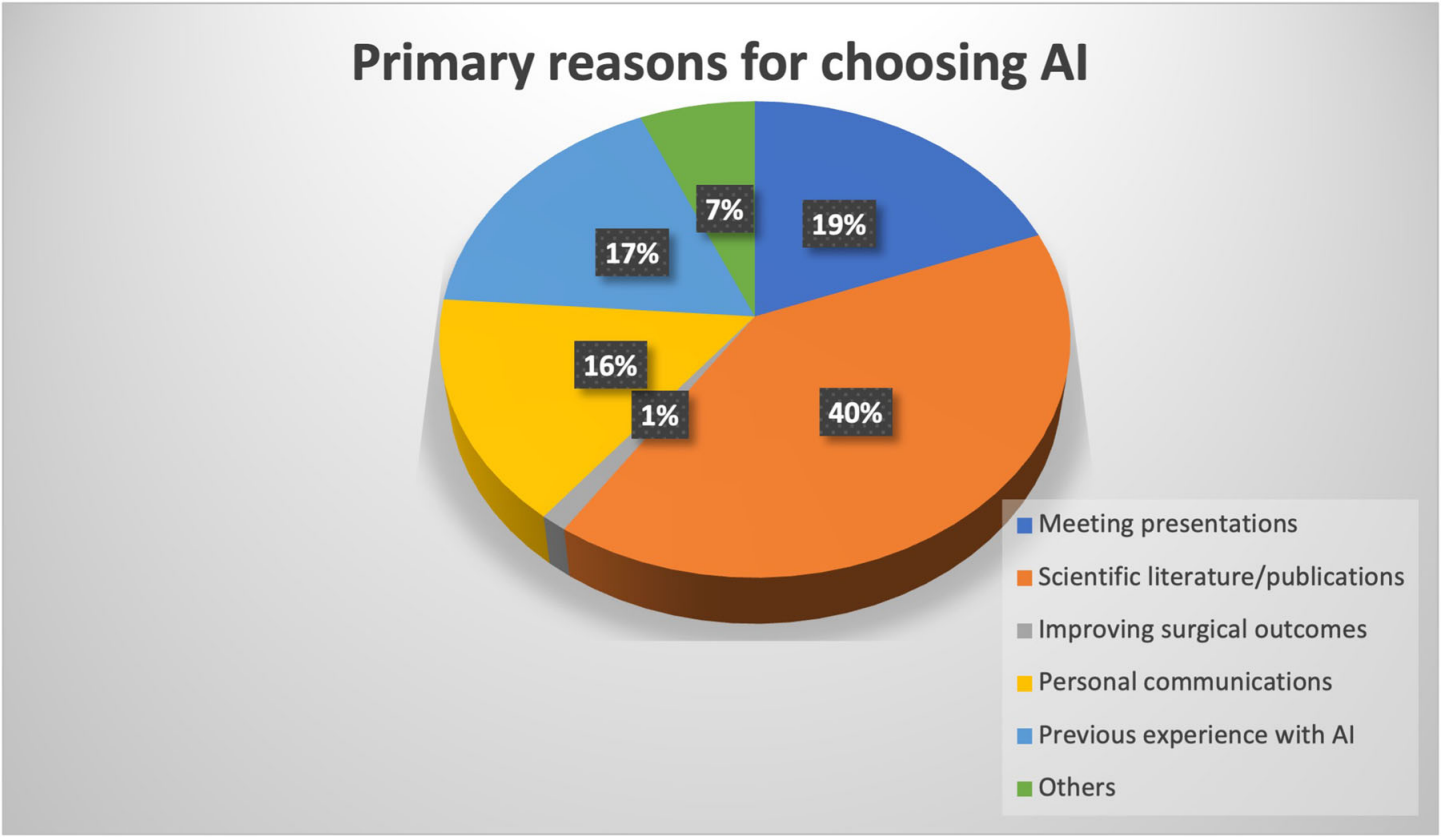
Pie chart depicting primary reasons survey participants use AI. AI, artificial intelligence.

A considerable proportion (25.1%) reported using AI in their practice, with primary reasons including referencing scientific literature/publications (40.3%) and engaging in personal communications (15.3%) (Figure 2). Interestingly, only a small percentage reported choosing AI to improve surgical outcomes (1.2%). Regarding the routine use of AI, a minority reported always or often utilizing AI for image‐based diagnosis (11.9%) and intraoperative aid (11.9%). Similarly, for robotic surgery, the minority reported always or often using AI (16.1%). When considering the use of big data, respondents expressed interest in leveraging it to improve diagnosis (30.3%) and treatment (47.4%). However, a small percentage expressed concerns about the potential worsening of diagnosis or treatment (1% each). Results are reported in Table 4.

### AI and its future

**Figure 3:**
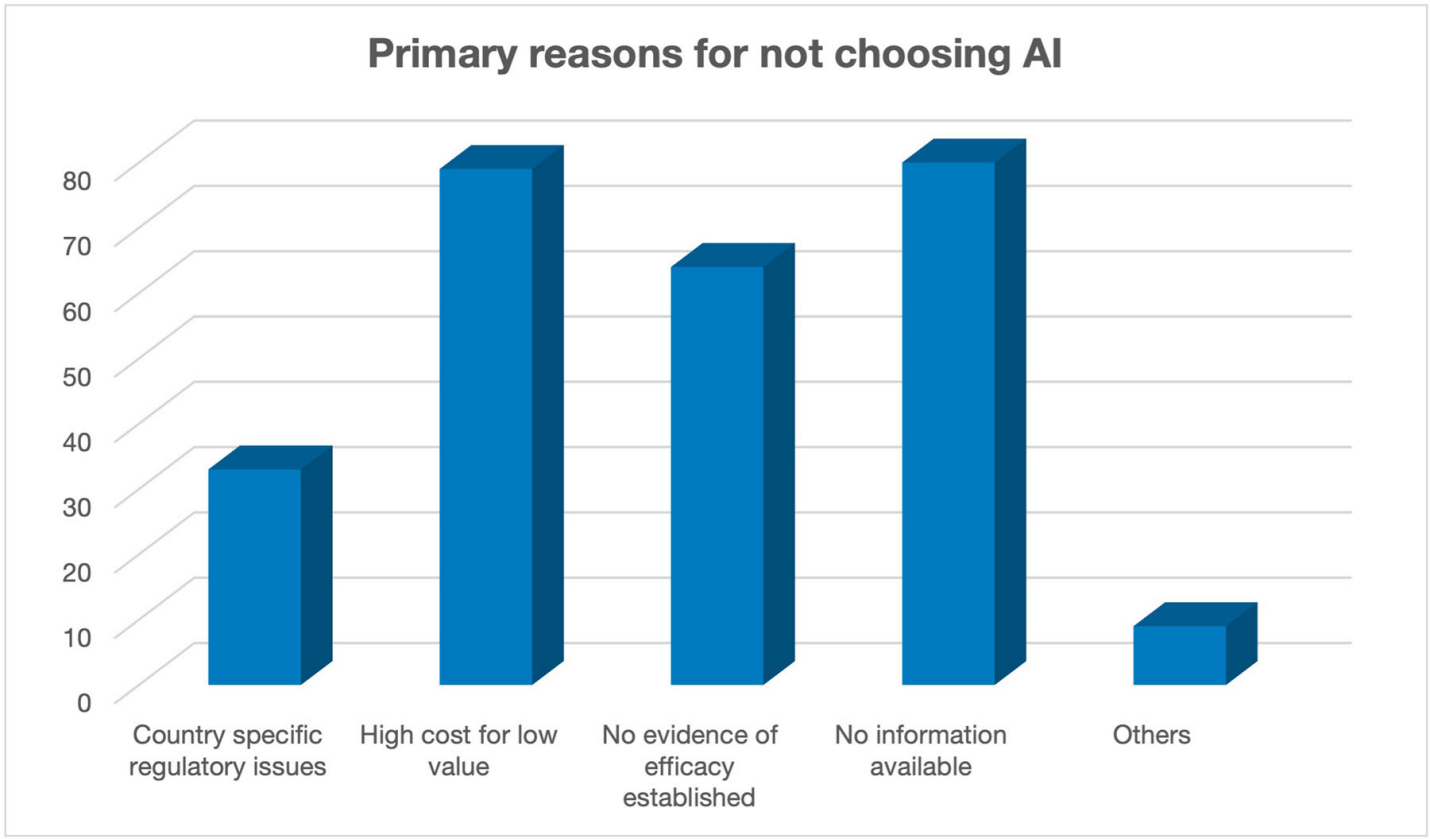
Figure depicting the most common reasons survey participants do not use AI. AI, artificial intelligence.

A vast majority expressed an interest in leveraging AI technologies (95.3%), demonstrating a keen inclination towards incorporating AI into orthopaedic practice. Regarding ML and DL, respondents indicated specific areas of interest for further study, including prediction of patient outcomes after surgery (30.8%) and an image‐based diagnosis of osteoarthritis (OA) (28%). Moreover, participants expressed preferences for AI technologies they would like to see in the future, with AI emerging as the top choice (60.7%), followed by DL (20.9%) and ML (15.2%). However, barriers to AI adoption were also identified, including country‐specific regulatory issues (12.5%), high cost for perceived low value (29.8%), and a lack of established efficacy evidence (24.2%) (Figure 3).

## DISCUSSION

This study presents the results from a survey conducted by a worldwide society (ISAKOS) about AI in orthopaedics. Previous surveys have been conducted in the orthopaedic field; however, they have not included all of these countries or have focused on a specific topic [3, 7, 11]. Most of the participants were male (92.9%), medical doctors (97.6%) and orthopaedic surgeons (63%) from all over the world. The most represented regions were Europe (37.9%) and Asia (25.6%).

The most important finding of this survey was that 74.9% of the participants do not use AI in their practices; this seems to be mainly due to a lack of information (30.2%), high cost (29.8%) and a lack of established evidence of efficacy (24.2%). The high cost of AI devices has previously been highlighted by Desai et al. [3]. The authors reported the findings of a survey on the use of AI in arthroplasty surgery to all the members of the Indian Society of Hip and Knee Surgeons (ISHKS). Of these, 417 responded to the questions, and for 93.52%, the high cost of robot installation was a limitation for its use. In addition, for 82.7%, another limitation was the lack of complete insurance coverage for robotic‐assisted arthroplasty procedures. Although the implementation of AI software is not always as expensive as robotics, the upfront cost of the software, the training required for the software and those using it, and the integration into clinical practice can be expensive and time‐consuming. Comparing these results to our population, a possible explanation could be that 45% of the participants work in private practice, 23.3% work in a public hospital and 31.3% hold an academic position.

The level of surgical training of respondents of this survey was high, as 52.6% of the participants have more than 20 years of experience. This also means that most participants in the survey were in the older group, which could be another explanation for the reluctance to include AI in their medical practice. However, Weidener and Fischer [15] reported that only 39% of medical students from Germany, Switzerland and Austria had prior experience with AI‐based chat applications for any use, such as ChatGPT, demonstrating that a reluctance to use AI might not be an age‐related issue and that the lack of experience could be unrelated to the clinical scenario but in general with AI itself. Of the students who had used AI‐based chat applications, 73% had used it for medical‐related contexts.

Another interesting finding was that a similar percentage of the participants knew (47.9%) and did not know (52.1%) the difference between AI, ML, and DL. However, they mostly did not know where and when AI, ML or DL are used in medicine (53.6%) or in orthopaedics (54.5%). Kamal et al. [7] published a survey focused on the evaluation of the perceptions, attitudes and interests of Sudanese orthopaedic surgeons regarding different applications of AI in orthopaedic surgery. They also assessed the knowledge of AI in their study, and they found that 54.7% of the participants did not know the difference between ML and DL and 74.2% did not know the difference between supervised and unsupervised ML. This reveals confusion and a lack of knowledge around this topic, which may also be related to the presently low use of AI.

In the present study, most participants agreed with the definitions given for AI (90.5%), ML (88.2%) and DL (81.5%), demonstrating that DL is probably the most unknown element. Nevertheless, the percentage of agreement with the definition was high. There were no alternative definitions, which could increase the percentage of agreement without meaning that there is a real understanding of each of them.

In this study, 24.2% of participants answered that the main reason they do not use AI was the lack of evidence of established efficacy. Luyckx et al. [11] published a survey of the members of the European Knee Society (EKS) and reported that 82% believe that robot‐assisted total knee arthroplasty (TKA) has a significant impact on the accuracy of bone cuts, 90% believe that robot‐assisted TKA helps to customize the alignment and 92% believe that this helps in the correct implant positioning. Nevertheless, there was no agreement on the peri‐ and post‐operative outcomes. Over the years, many studies have focused on post‐operative outcomes after conventional arthroplasty and robot‐assisted arthroplasty, but it has been reported that there are no differences in functional outcomes between these two techniques [2, 12].

Looking to the future, 30.8% agreed that it would be useful to improve studying ML and DL to predict post‐operative outcomes. This purpose has often been the subject of research, but it is still not possible to use it routinely due to the current poor accuracy, and further research is needed in this direction [5, 6]. In the present study, 28% of the participants proposed to study more ML and DL to increase the image‐based diagnosis of OA. Swiecicki et al. [13] proposed an automated DL‐based algorithm through the posteroanterior and lateral projections of knee radiographs can assess the severity of knee osteoarthritis according to the Kellgren–Lawrence classification system with optimal results; they demonstrated that this algorithm could provide an accurate and reproducible measure of osteoarthritis severity for research and clinical decision‐making.

This survey has some limitations. First, the majority of participants were ISAKOS members; this could represent a bias because of the particular interest of this group of people. This survey was also not pretested or tested for reliability. Another limitation was that the responses were provided primarily by participants from Europe and Asia, which may limit the extrapolation of the results. Additionally, over 90% of the respondents were male, which could induce some sex‐based bias. Finally, as the questions were asked online, this could result in a lower understanding of all the questions and therefore less accurate answers, but also a lack of discussion of their answers.

## CONCLUSION

This survey demonstrates that there is currently limited use of AI in orthopaedic practice, mainly due to a lack of knowledge about the subject, a lack of proven evidence of its real utility and high costs. However, there is a high level of interest in its use in the future, and an increase in study and research on the subject is required so that it can be of real benefit and make AI an integral part of the orthopaedic surgeon's daily work.

## LIST OF COLLABORATOR

The ISAKOS Young Professionals Task Margaret W. M. Fok, FRCSEd(Ortho), MBChB, Camilo P. Helito, MD, PhD, Michael J Alaia, MD, Hamid Rahmatullah Bin Abd Razak, MBBS, FRCSEd (Ortho), FRCSGlasg (Tr & Orth), FAMS, Riccardo Cristiani, MD, PhD, Peter Alberto D Alessandro, MBBS Hons. (UWA) FRACS FAOrthA, Franco Della Vedova, MD, Rachel M. Frank, MD, Mohamed A. Imam, MD, MSc, DSportMed, ELD (Oxon), PhD, FRCS, Nikolaos K. Paschos, MD, PhD, Simone Perelli, MD,PhD, Sven Edward Putnis, MB ChB FRCS(Tr&Orth), Ross Radic, MBBS FRACS (Ortho) FAOrthA, Luís Duarte Silva, MD, Gustavo Vinagre, MD, PhD, Megan R. Wolf, MD.

## AUTHOR CONTRIBUTIONS

Filippo Familiari, Juan Pablo Martinez‐Cano, Juan Miguel Del Castillo, Nicholas N. DePhillipo, Gilbert Moatshe, Edoardo Monaco and Jaime Palos Lucio contributed to the study design, data collection and manuscript preparation. Filippo Familiari, Juan Pablo Martinez‐Cano, Juan Miguel Del Castillo, Nicholas N. DePhillipo, Gilbert Moatshe, Edoardo Monaco and Jaime Palos Lucio contributed to data analysis and manuscript review. Adnan Saithna, Jorge Chahla, Pieter D'Hooghe and Robert F. LaPrade provided clinical input and manuscript editing. All authors supervised the research and approved the final manuscript.

## ETHICS STATEMENT

The authors have nothing to report.

## CONFLICT OF INTEREST STATEMENT

The authors declare no conflicts of interest.

## Data Availability

The data that support the findings of this study are available upon request from the corresponding author.
